# A real-world experience of efficacy and safety of belantamab mafodotin in relapsed refractory multiple myeloma

**DOI:** 10.1038/s41408-025-01226-8

**Published:** 2025-03-10

**Authors:** Rachel Dileo, Prerna Mewawalla, Kalaivani Babu, Yue Yin, Christopher Strouse, Ethan Chen, Hira Shaikh, James A. Davis, Kimberly M. Green, Omar Alkharabsheh, Aliya Rashid, Bidushi Pokhrel, Nausheen Ahmed, Al-Ola Abdallah, Hamza Hashmi

**Affiliations:** 1 Division of Hematology and Cellular Therapy, Allegheny Health Network, Pittsburgh, PA 15222 USA; 2 Allegheny-Singer Research Institute, Allegheny Health Network, Pittsburgh, PA USA; 3 Division of Hematology, Oncology, and Blood and Marrow Transplantation, Holden Comprehensive Cancer Center, University of Iowa Hospital and Clinics, Iowa City, IA USA; 4 Department of Hematology-Oncology, Medical University of South Carolina, Charleston, SC USA; 5 University of South Alabama Mitchell Cancer Institute, Mobile, AL USA; 6 Division of Hematologic Malignancies & Cellular Therapeutics, University of Kansas Cancer Center, Kansas City, MO USA; 7 Myeloma & Cell Therapy Service, Memorial Sloan Kettering Cancer Center, New York, NY USA

**Keywords:** Cancer immunotherapy, Myeloma

## Abstract

While initial trials led to the accelerated approval of belantamab mafodotin, a BCMA-directed antibody-drug conjugate, confirmatory trials failed to establish benefit from this therapy for patients with relapsed refractory multiple myeloma (RRMM), eventually leading to its withdrawal from commercial use. With an imminent approval as an effective combination therapy, as seen in recent randomized trials, we report real-world clinical outcomes with belantamab mafodotin in 81 RRMM patients. With a median of 5 (range 2–15) prior lines of therapy, 92, 45, and 15% of the patients were triple-class refractory, penta-class refractory, and BCMA-refractory. More than half (57%) of the patients had high-risk cytogenetics, 37% had extramedullary disease (EMD), and 67% of the patients would have been considered ineligible for the DREAMM-2 trial. The best overall response (ORR) and complete response rates were 40.0 and 15.0%, respectively. ORRs were lower in patients with EMD, BCMA-refractory, and penta-refractory disease at 23, 17, and 24%, respectively. All-grade ocular toxicity was seen in 69% of patients, with grade 3+ events in 43%. Grade 3+ hematological toxicities included neutropenia (20%), anemia (28%), and thrombocytopenia (31%). With a median follow-up of 11.3 (0.3–44.6) months for the entire population, median PFS and OS were 5 (1–20) months and 12 (3–28) months, respectively. Presence of EMD was the only predictor of both PFS and OS on multivariable analysis. Compared to the pivotal trial and despite several high-risk disease features, belantamab mafodotin demonstrated comparable efficacy and safety in this real-world patient population.

Multiple myeloma (MM) is an inherently incurable plasma cell malignancy characterized by periods of prolonged remissions and inevitable relapses. With a median progression-free survival (PFS) of 3 to 4 months and an overall survival (OS) of less than 12 months, triple-class refractory patients represent a significant unmet need in the treatment landscape of relapsed refractory multiple myeloma (RRMM) [1]. With its uniform expression and pivotal role in plasma cell proliferation, B-cell maturation antigen (BCMA) represents an ideal therapeutic target for an antibody-drug conjugate (ADC) known as belantamab mafodotin [2, 3] Despite the initial accelerated approval as monotherapy for RRMM patients who had failed ≥4 prior lines of therapy (LOT), subsequent randomized confirmatory trial (DREAMM-3) failed to demonstrate statistically significant improvement in outcomes, leading to the withdrawal of belantamab mafodotin from the US market in November 2022 [4, 5]. However, data from recent phase III randomized controlled trials (DREAMM-7 and DREAMM-8) [6, 7] demonstrated significant improvements in depth and duration of response with belantamab mafodotin combination therapies when compared to standard-of- care treatments for RRMM. With the imminent return of belantamab mafodotin to the U.S. market and expected utilization in clinical practice, we report the real-world safety and efficacy outcomes of standard-of-care belantamab mafodotin for RRMM, with the aim to determine the patterns of its use in a real-world population and highlight the outcomes in high-risk patients who would have been otherwise considered ineligible to receive belantamab mafodotin in a clinical trial.

In this study, we conducted a retrospective chart review of adult patients with RRMM who were treated with belantamab mafodotin monotherapy under the FDA-approved label from October 10, 2019 till September 28, 2023. A total of 81 patients with RRMM were included in the analysis, with baseline patient and disease characteristics highlighted in Table 1. The median age of the entire patient population was 67 years (range 37–85), with 63% of the patients being male. With a median of 5 (range 2–10) prior lines of therapy, 92, 45, and 15% of the patients were triple-class refractory, penta-class refractory, and BCMA-refractory, respectively. More than half (57%) of the patients had high-risk cytogenetics, 37% had extramedullary disease (EMD), 37% had an Eastern Cooperative Oncology Group (ECOG) performance status (PS) of 2–4, and 67% of the patients would have been considered ineligible for the DREAMM-2 trial.

**Table 1:** Baseline patient and disease characteristics.

Characteristic	*N* (%)
Male	51/81 (63)
Female	30/81 (37)
Age median (range)	67 (37–85)
Age >70 years	34/81 (42)
Race, *n* (%)	
Non-Hispanic White	62 (77)
Non-Hispanic Black	14 (17)
Asian	1 (2)
Hispanic	3 (4)
Cardiac dysfunction (LVEF <40%), *n* (%)	11/81 (14)
Renal dysfunction (GFR <60 mL/min), *n* (%)	35/81 (47)
Lung dysfunction (COPD or asthma), *n* (%)	10/81 (12)
Baseline cytopenia, grade 3+, *n* (%)	19/81 (23)
IgG subtype, *n* (%)	44/81 (54)
R-ISS stage	
Stage I, (%)	14/67 (21)
Stage II, (%)	22/67 (33)
Stage III, (%)	31/67 (46)
ECOG PS, *n* (%)	
0–1	51/81 (63)
2–4	30/81 (37)
High-risk cytogenetics *n* (%)	
Del(17p)	12/81 (15)
t(4;14)	14/81 (17)
t(14;16)	1/81 (1)
gain/amp 1q	33/81(41)
EMD, *n* (%)	30/81(37)
Prior LOT, median (range)	5 (2–10)
≥4 LOT, *n* (%)	65/81 (80)
Refractory status *n* (%)	
PI	73/81 (91)
IMiD	76/81 (94)
BCMA	12/81 (15)
BsAB	7/12 (58)
CAR T	3/12 (25)
Trispecific T-cell engager (HPN217)	2/12 (17)
Double-refractory	77/81 (95)
Triple-refractory	74/81 (91)
Penta-refractory	37/81 (46)
Prior SCT, *n*(%)	
Autologous	55/81 (68)
Allogeneic	2/81 (3)

*Amp* amplification, *BCMA* B-cell maturation antigen, *BsAB* bispecific antibodies, *CAR T* chimeric antigen receptor T-cell therapy, *COPD* chronic obstructive pulmonary disease, *ECOG* eastern cooperative oncology group, *PS* performance status, *EMD* extramedullary disease, *Del* deletion, *GFR* glomerular filtration rate, *IMiD* immunomodulatory drugs, *LOT* lines of therapy, *LVEF* left ventricular ejection fraction, *PI* proteasome inhibitors, *R-ISS* revised international staging system, *SCT* stem cell transplant.

With a median follow-up of 11.3 (range 0.3–44.6) months and median number of six cycles (range 1–15) for the entire patient population, best ORR was 40% with 15% of the patients achieving a complete response or better. Partial response, very good partial response, and stable disease were seen in 17, 7, and 23% of the patients, respectively. In comparison, patients with BCMA-refractory disease, penta- refractory disease, and EMD had the best ORR of 17, 24, and 23%, respectively. The median time to first response was 21 days (range 20–42), and the median time to best response was 42 days (range 21–80). Of the 32 patients that responded, the median duration of response was 13 months (range 10.1–26.9). Median PFS was 5 months (range 1–20), and median OS was 12 months (range 3–28) for the entire patient population (Fig. 1A, B). For patients with high-risk cytogenetics, ECOG 2–4, and EMD at the time of treatment; median PFS was 5 (range 1–12), 3 (range 1–16), and 2 (range 1–5) months, respectively. Similarly, for patients with high-risk cytogenetics, ECOG 2–4, and EMD at the time of treatment; median OS was 9 (range 2–22), 5 (range 2–21), and 5 (range 2–15) months, respectively (Fig. 1C–H). Using the cox proportional hazard model, the presence of EMD was the only clinical variable associated with inferior PFS (HR = 1.97, 95% CI [1.11, 3.50], *p* = 0.0205) as well as OS (HR = 3.05, 95% CI [1.71, 5.45], *p* = 0.0002). At the time of the last follow-up, 58 (72%) patients had experienced disease progression on belantamab mafodotin with most common salvage therapies including alkylating agents (ten patients, 23%), selinexor combinations (eight patients, 19%), and T-cell-directed therapies (seven patients,16%), respectively.

**Fig. 1:**
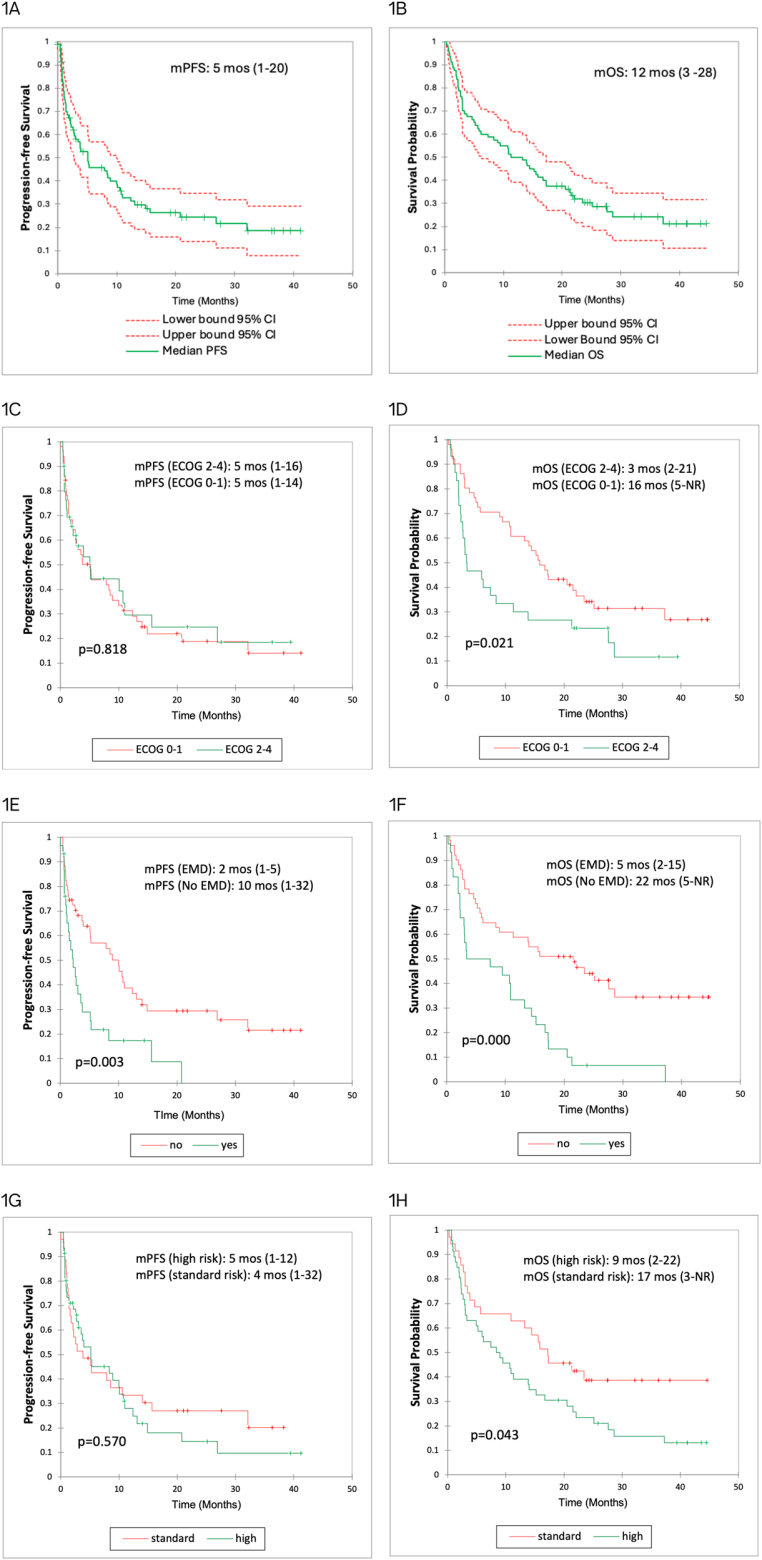
PFS and OS Analysis. **A**, **B** Kaplan–Meier curves of median PFS and OS for all patients. **C**, **D** Kaplan–Meier curves of PFS and OS for patients with high-risk versus standard risk cytogenetics. **E**, **F** Kaplan–Meier curves of PFS and OS for patients with ECOG PS 0–1 versus 2–4. **G**, **H** Kaplan–Meier curves of PFS and OS for patients with EMD versus no EMD. PFS progression-free survival, OS overall survival, m median, EMD extramedullary disease, ECOG PS eastern cooperative oncology group performance status, NR not reached.

Regarding ocular toxicity, any grade keratopathy was seen in 56 (69%) patients, with 24 (30%) patients experiencing grade 3 or worse events. The median time to onset of ocular toxicity was 21 days (range 15–98), and the median time to improvement to grade I or complete resolution was 42 (21–180) days. Twenty-six patients (32%) had a visual decline of <20/50 and 17 patients (21%) had a visual decline of <20/200 on the visual acuity scale. A total of 59, 36, and 53% of the patients had dose delay/interruption, dose reduction, and treatment discontinuation, respectively. Furthermore, 18 (22%) patients had greater than 12 consecutive weeks of treatment delay. For hematologic toxicities, 28 (35%), 16 (20%), 23 (28%), and 25 (31%) patients developed grade 3+ leukopenia, neutropenia, anemia, and thrombocytopenia, respectively. More than one-quarter (27%) of the patients experienced an infection, with 17% of all patients developing a severe infection (grade ≥3). At the time of last follow-up, 60 (74%) patients had died with 68, 13, and 12% of these deaths related to disease progression, infection, and organ failure, respectively.

To our knowledge, this multicenter retrospective study is one of the largest real-world reports comprehensively describing clinical outcomes in RRMM patients treated with standard-of-care belantamab mafodotin. In our analysis, this therapy was associated with notable efficacy in patients with heavily pretreated disease who had progressed through multiple LOT, including prior BCMA-directed therapy. There are some reports of real-world experience with belantamab mafodotin, however, these studies are either limited by the small number of patients [8–10] or exclusion of real-world patients under the strict criteria of compassionate use [11]. While a large study has reported outcomes of belantamab mafodotin in RRMM [12], our analysis had higher a proportion of high-risk patients with triple and penta-class refractory disease as well as EMD for a comprehensive subgroup analysis to determine clinical predictors of efficacy. While the toxicities noted were similar to the previously published clinical trials, two-third of the patients in our real-world analysis would have been considered ineligible for the DREAMM-2 trial due to severe baseline cytopenias (24%), renal insufficiency (43%), and ECOG PS 3–4 (5%). Despite these differences, response rates in our study were similar to the DREAMM-2 trial patients (ORR 40% versus (vs) 31%; PFS 5 months vs 2.9 months). More importantly, there was no significant increase in the risk of severe ocular (30 vs 27%), and hematologic toxicities, i.e., thrombocytopenia (31 vs 20%) and anemia (28 vs 20%), despite the presence of severe baseline cytopenias and impaired end organ function [13]. It is important to highlight that the frailty, cytopenias, and organ dysfunction seen in these patients at the time of relapse are often related to prior therapy and disease progression. These patients reflect a real-world population in need of effective treatments, and the presence of one or more of these clinical trial exclusion criteria should not be conceived as a barrier to receiving effective salvage therapies, especially if these are considered safe with manageable toxicities. Similar to published literature, the most common toxicities associated with belantamab mafodotin included keratopathy and decline in visual acuity, with more than two-third of the patients developing ocular toxicities. Keratopathy resolved in a majority of the patients, with a median time to resolution of 6 weeks, and no patients developed permanent blindness. However, this toxicity did lead to more than half (53%) of the patients discontinuing treatment, likely due to the impact on quality of life, raising the need for less frequent dosing. Presence of EMD was a predictor of both inferior PFS as well as OS on multivariable analysis. EMD is considered a high-risk disease feature for RRMM and has been associated with early progression and overall poor prognosis after BCMA-directed and other traditional plasma cell-directed therapies [14, 15]. Tumor-driven inhibition of the immune microenvironment likely contributes to the effector and target disparity, because large tumors seen in EMD require more robust activation of host immune effector cells for deep and durable responses. Consideration should be given to combination therapies, including belantamab with pomalidomide/bortezomib and dexamethasone or administering concurrent radiation therapy to enhance the tumoricidal effect. While our study had a small number of patients with exposure to prior BCMA-directed therapy; all of these patients were refractory to this class of drug, and the majority had received BCMA-directed monoclonal antibody. The poor efficacy seen in this subgroup could be related to loss/ downregulation of target antigen or T-cell exhaustion with the ongoing treatment with monoclonal antibody and T-cell engagers. Whether patients with late relapse after CAR T still may be good candidates for retargeting of BCMA with belantamab mafodotin remains an important clinical investigation.

In summary, belantamab mafodotin appeared safe and relatively effective in this real-world heavily pretreated RRMM population, where the majority of patients did not meet the eligibility criteria for the DREAMM-2 trial and had high-risk disease features. Ocular toxicities remained a major therapeutic challenge, highlighting the need for future investigations to optimize dosing and frequency of administration for better tolerability.

## Data Availability

The data that support the findings of this study are available from the corresponding author upon reasonable request.
